# Thermionic Electron Beam Current and Accelerating Voltage Controller for Gas Ion Sources

**DOI:** 10.3390/s21082878

**Published:** 2021-04-20

**Authors:** Jarosław Sikora, Bartosz Kania, Janusz Mroczka

**Affiliations:** 1 Department of Automatics and Metrology, Lublin University of Technology, 20-618 Lublin, Poland; jaroslaw.sikora@pollub.pl; 2 Department of Electronic and Photonic Metrology, Wroclaw University of Science and Technology, 50-370 Wroclaw, Poland; janusz.mroczka@pwr.wroc.pl

**Keywords:** thermionic emission, electron accelerating voltage, electron beam current, ion current, controller, standard deviation

## Abstract

Thermionic emission sources are key components of electron impact gas ion sources used in measuring instruments, such as mass spectrometers, ionization gauges, and apparatus for ionization cross-section measurements. The repeatability of the measurements taken with such instruments depends on the stability of the ion current, which is a function, among other things, of the electron beam current and electron accelerating voltage. In this paper, a laboratory thermionic electron beam current and accelerating voltage controller is presented, based on digital algorithm implementation. The average value of the percentage standard deviation of the emission current is 0.021%, and the maximum electron accelerating voltage change versus the emission current is smaller than 0.011% in the full operating range of the emission current. Its application as a trap current or emission current-regulated ion source power supply could be useful in many measuring instruments, such as in microelectromechanical system (MEMS) mass spectrometers as universal gas sensors, where a stable emission current and electron energy are needed.

## 1. Introduction

The thermionic electron sources operating in the Schottky current range used in gas ion sources generate an ionizing electron beam with a specific intensity and energy. They are widely used in many measuring tools, such as apparatus for ionization cross-section measurements [1,2,3], ionization gauges [4,5], mass spectrometers, and in microelectromechanical systems (MEMS) technology [6,7]. Electron beam ion source technology has been applied in the Relativistic Heavy Ion Collider at Brookhaven National Laboratory [8].

The measure of the electron ionization process is the ion current *I* directly proportional to the concentration of the gas molecules. Assuming that all ions produced in the ion source are extracted from the source, the expression of *I* can be written as follows:

(1) $$I=nI_{e}lQ\left(E\right)$$

(2) E=eV

where *n* is the concentration of the gas molecules; *I_e_* is the ionizing electron thermionic emission current, hereinafter referred to as the emission current or electron beam current; *l* is the effective ionizing path length; *Q*(*E*) is the total electron impact ionization cross-section function of the electron energy *E*; *e* is the electron charge; and *V* is the electron accelerating voltage. It was assumed that the initial energy of emitted electrons is negligible. 

In ion source operating conditions, the emission current significantly depends on the changes in the cathode work function caused by adsorption-related phenomena on its surface. A method of dynamic measurement of work function variations of metal cathodes caused by adsorption of residual gases and results for tungsten and tantalum cathodes are presented in paper [9]. Variations of the electron beam current affect the stability of the ion current and decrease the repeatability of measurement results of a gas concentration or ionization cross-section (see Equation (1)). However, as the ratio of the ion current over the electron beam current is measured, the need for electron beam current stabilization can be eliminated, for example, in cross-section measurements [1,2,3]. In ionization gauges and mass spectrometers, for the high stability of sensitivity and repeatability of measurement results [4,5], the electron beam current and accelerating voltage should be maintained at preset constant values, and, therefore, both quantities must be perfectly stabilized, which complies with the concept of the cognitive process in metrology [10]. It should be noted that, in some instruments, such as mass spectrometers, the Klopfer ionization gauge [5], only a part of the electron beam current collected by a trap electrode, as a true ionizing current, should be stabilized [11].

Many control systems of thermionic electron sources established upon analogue [11,12,13,14,15,16,17,18,19] or digital [20,21] technology provide only emission or the trap current stabilization. The electron accelerating voltage is dependent on the cathode heating voltage and the voltage drop across the emission current sensing resistor (see Equation (3)). For this reason, the selection of optimal electron impact ionization conditions is difficult and time-consuming. Several circuits ensure the independence of the electron accelerating voltage from the voltage drop across the electron emission sensing resistor, but the effect of the cathode heating voltage is not reduced [19,22,23]. In the digital controller of the ionization vacuum gauge [24], to control the electron accelerating voltage, the anode voltage and the cathode biasing voltage are stabilized, but the influence of the cathode heating voltage still remains. The circuit presented in paper [25] allows independent setting and stabilization of the emission current and the electron accelerating voltage, but it is made with analogue technology without the abilities of proportional–integral–derivative (PID) controller tuning, system management, and monitoring.

This work describes a digital control system of thermionic electron sources operating in gas ion sources, which ensures the independent control of the electron beam current and accelerating voltage. The controller was developed to work with hot cathode electron sources with a heating current of up to 3 A, an emission current of up to 16 mA, and an accelerating voltage of up to 125 V.

## 2. Design

**Figure 1:**
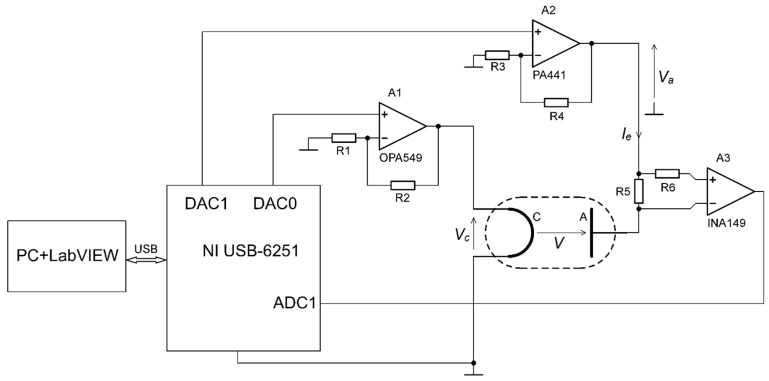
A simplified diagram of the control system electrical circuit. *V_c_* is the cathode voltage, *I_e_* is the emission current, *V_a_* is the anode circuit supply voltage, *V* is the electron accelerating voltage. *R*_1_ = 12 kOhm; *R*_2_ = 2.4 kOhm; *R*_3_ = 10 kOhm; *R*_4_ = 120 kOhm; *R*_5_ = *R*_6_ = 570 Ohm. The operational amplifier A_1_ and differential amplifier A_3_ are supplied from the voltage source of +/−12 V/5 A, and the operational amplifier A_2_ is supplied from the voltage source of 125 V/100 mA. All supplied voltage sources are referenced to the ground.

A simplified diagram of the control system electrical circuit is shown in Figure 1.

The control system consists of a National Instruments USB data acquisition card (NI USB-6251), a personal computer with Windows and LabVIEW authors software, the controlled system, and intermediary circuits. The data acquisition card uses a 16-bit, 1 MS/s analogue-to-digital converter and two 16-bit, 2.8 MS/s digital-to-analogue converters and is responsible for signal conversion between the control algorithm and the system. 

The voltage drop that is developed across the R_5_ resistor by the flowing emission current is transferred as a negative feedback signal from the high-voltage anode circuit to the low-voltage cathode circuit by a precision unity-gain difference amplifier A_3_ (INA149 from Texas Instruments). The high-current operational amplifier A_1_ (OPA549 from Texas Instruments) controlled by the DAC0 card output voltage drives the cathode in order to obtain the desired emission current *I_e_*.

The high-voltage power operational amplifier A_2_ (PA441 from Apex Microtechnology) is controlled by DAC1 card output voltage. Its output voltage serves to supply the anode circuit. According to the results shown in Figure 1, the electron accelerating voltage may be written in the following form:

(3) $$V=V_{a}-I_{e}R_{5}-0.5V_{c}$$

According to above equation, the control system, for each value of the emission current *I_e_* and the cathode voltage *V_c_* over the whole operating range, automatically adjusts the anode circuit supply voltage *V_a_* to maintain a fixed value of the electron accelerating voltage, *V* and, finally, electron energy, *E* (see Equation (2)). Therefore, the proposed control system gives a precise value of the electron accelerating voltage, which is usually omitted in other reported circuits [11,12,13,14,15,16,17,18,19,20,21,22,23,24]. 

**Figure 2:**
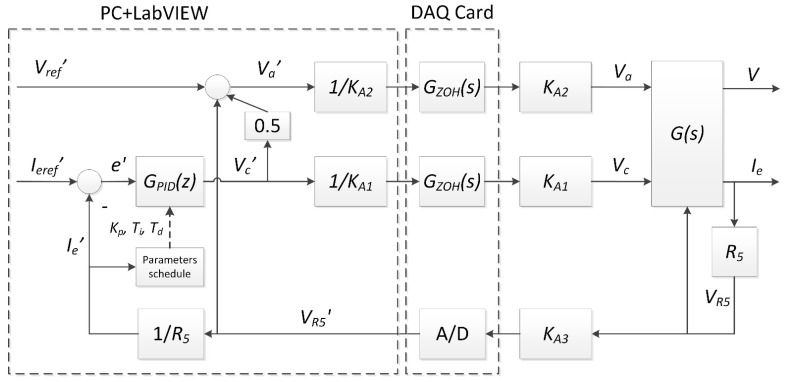
A block diagram of the designed control system.

A block diagram of the designed control system is presented in Figure 2, where *I_eref_’* is the discrete reference value of the emission current, *e’* is the error, *V_c_’* is the discrete value of the output voltage of the control algorithm, *V_R_*_5_ is the feedback voltage (the voltage drop across R_5_ resistance) proportional to the system output value *I_e_*, and *V_R_*_5*′*_ is the digital value of the feedback voltage for an emission current control loop. *I_e_’* is the digital representation of the emission current *I_e_*. *K_p_*, *T_i_*, and *T_d_* are the controller tuning values, and they are selected according to the emission current and a tuning values map. *V_ref_’* is the digital value of the reference accelerating voltage, and *V_a_’* is the digital value of the anode supply voltage. *G_PID_(z)* is the discrete transfer function of the control algorithm; *G_ZOH_(s)* is the transfer function of the zero-order hold; *K_A_*_1_ = 1.2 V/V, *K_A_*_2_ = 13 V/V, and *K_A_*_3_ = 1 V/V are the pure gains of A_1_, A_2_, and A_3_ amplifiers, respectively; and *G(s)* is the transfer function of the thermionic electron source. The block named A/D realizes digital-to-analogue conversion. 

The thermionic electron source is described by nonlinear static characteristics [26], and its dynamic properties can be expressed by first-order inertia with delay [17]. For this reason, a modified three-input and two-output signal transfer function equation of the thermionic electron source can be written as follows:

(4) $$\left[\begin{array}{c} I_{e}\\ V \end{array}\right]=\left[\begin{array}{ccc} \frac{K(I_{e})}{T(I_{e})s+1}e^{-sT_{0}(I_{e})} & 0 & 0\\ -0.5 & 1 & -1 \end{array}\right]\left[\begin{array}{c} V_{c}\\ V_{a}\\ V_{R5} \end{array}\right]$$

where *K(I_e_)* is the gain, *T(I_e_)* is the time constant, and *T*_0_*(I_e_)* is the time delay dependent on the output value *I_e_* [26]. The *V_R_*_5_ value, as shown in Figure 1, is a signal that decreases the electron accelerating voltage *V*.

Due to the nonlinear character of the electron source, the nonlinear form of the PID controller, *G_PID_(z)*, is implemented:

(5) $$G_{PID}\left(z\right)=K_{p}\left(I_{e}\right)\left(1+\frac{T_{s}}{T_{i}\left(I_{e}\right)}\left[\frac{z+1}{z-1}\right]+\frac{T_{{}_{d}}\left(I_{e}\right)}{T_{s}}\left[\frac{z-1}{z}\right]\right)$$

where *K_p_(I_e_)* is the proportional gain, *T_i_(I_e_)* is the integral time coefficient, *T_d_(I_e_)* is the derivative time coefficient, and *T_s_* is the sampling period. The tuning parameters are dependent on the output value, *I_e_*. 

**Figure 3:**
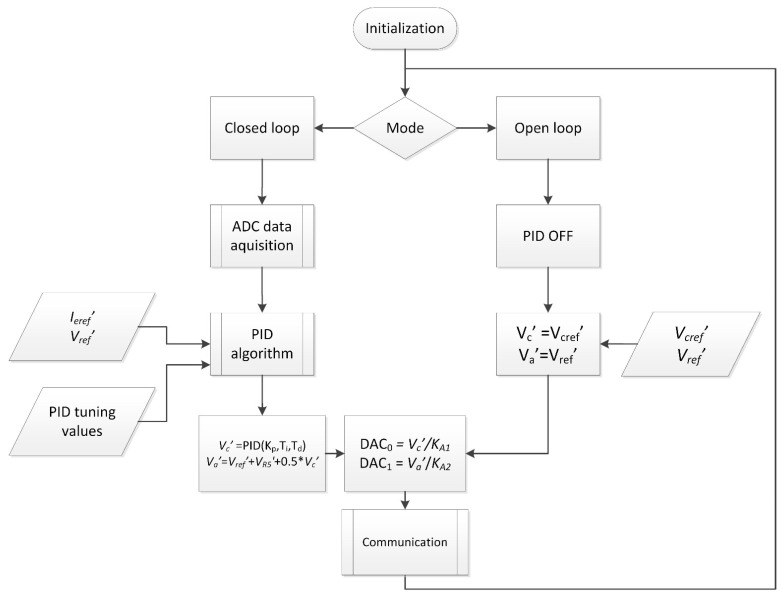
The general algorithm of the presented control system.

A general algorithm of the authors’ LabVIEW software is presented in Figure 3. It realizes proportional–integral–differential (PID) control to stabilize the electron beam current and a feedforward algorithm to make the electron energy and the electron beam current independently. The program applies the algorithm, which includes two modes of work: an open and closed loop. In the open-loop mode, the user determines only the cathode heating voltage *V_cref_’* and the accelerating voltage *V_ref_’*. These values are used as the heating voltage *V_c_’* and the anode supply voltage *V_a_’*. In the closed-loop mode, the voltage *V_R_*_5_ proportional to the emission current *I_e_* is acquired, averaged, and recalculated to the discrete emission current value *I_e_’*. Next, the discrete proportional–integral–derivative algorithm of the control is realized using PID tuning values dependent on the real value of the emission current to determine the heating voltage. The accelerating voltage is determined with the feedforward method using known values of the feedback voltage *V_R_*_5*′*_ and previously calculated heating voltage *V_c_’*. The last part of the control loop sets the output values of the controller.

## 3. Results

**Figure 4:**
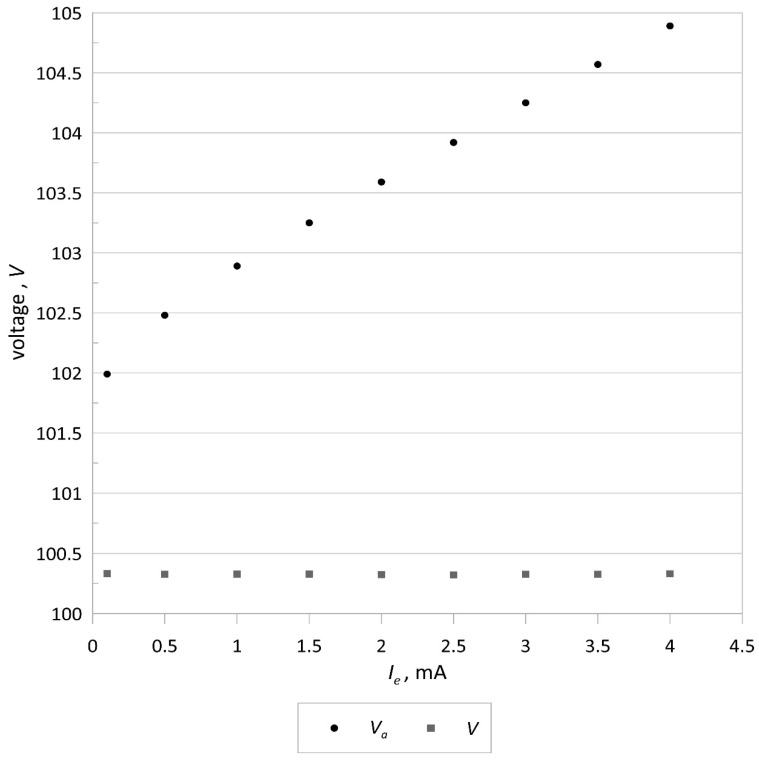
Results of the feedforward control of the electron accelerating voltage *V*.

**Figure 5:**
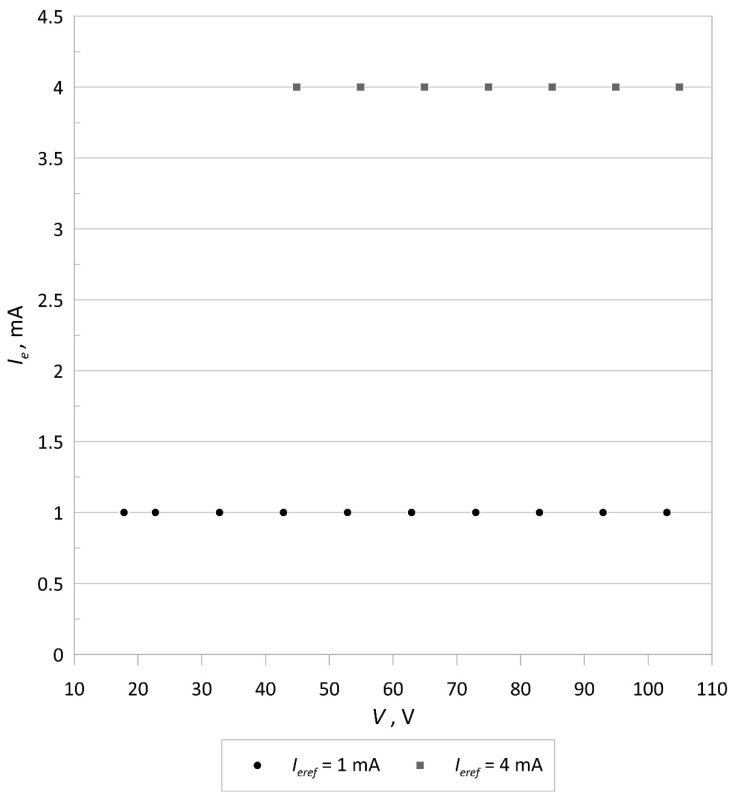
Plot of the emission current *I_e_* versus the electron accelerating voltage *V*.

The measurements of the emission current and the electron accelerating voltage were made for the electron source with a tungsten cathode (40 mm long, 0.1 mm in diameter) installed in a Bayard-Alpert gauge using HP 34461A multimeters. The total measurement errors of current intensity and voltage (including the reading error and the range error) are less than 0.027 and 0.0036%, respectively. Figure 4 shows the results of implementing feedforward control of the electron accelerating voltage, and Figure 5 presents the dependence of the emission current on the electron accelerating voltage *V*. The maximum percentage change in the electron accelerating voltage over the whole range of the emission current is smaller than 0.011%, which confirms that the implemented feedforward algorithm strongly reduces changes in the accelerating voltage during the emission current adjustment. This allows the electron energy, associated with the maximum value of the ionization cross-section *Q*, to be kept constant in the gas ion source while selecting the emission current to obtain satisfactory ionization efficiency. This property is also highly suitable to realize harmonic or pulse electron impact gas ionization at the given optimal electron energy. On the other hand, the experimental data presented in Figure 5 show that the emission current stays constant within a wide range of accelerating voltages, thereby confirming the proper operation of the control system as the accelerating voltage changes.

**Figure 6:**
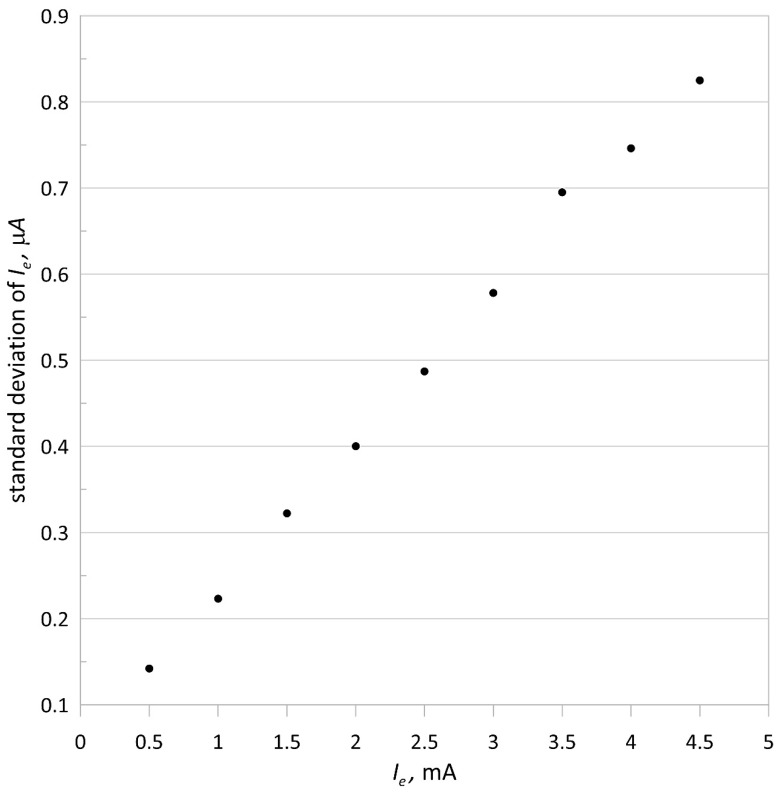
Standard deviation of the emission current versus its intensity; the accelerating voltage *V* = 100 V.

**Figure 7:**
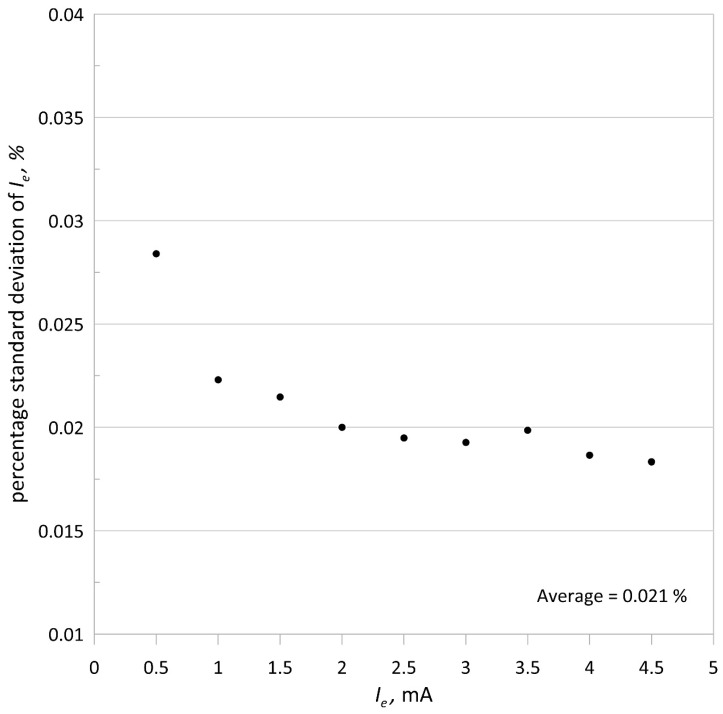
Percentage standard deviation of the emission current versus its intensity.

To show the stability of the emission current, its standard deviation was determined. The duration of the measurement for each value of the emission current was 180 s, which is longer than the usual measurement time for mass spectrometer isotope ratios. Figure 6 and Figure 7 show plots of the standard deviation and percentage standard deviation of the emission current, respectively.

The average value of the percentage standard deviation of the emission current is 0.021%, and the maximum percentage change in the electron accelerating voltage is smaller than 0.011%, indicating the high quality of the ionizing electron beam. The obtained two-hour standard deviation for 1 mA of the emission current and 100 V of the accelerating voltage was equal 225 nA (0.025%), which is comparable to the 180 s emission current standard deviation.

The presented system represents the next step after the simple digital thermionic PID controller described in [21]. Table 1 contains a comparison of both systems.

The proposed system is lab-friendly because it uses high-level programming language, a PC, a data acquisition card, and off-the-shelf components, which allows for the system to be easily built, run, and tested. Such a system also allows one to acquire the electron emission current, the electron accelerating voltage, and their statistics parameters. The current form of the control system is relatively expensive, but it can be realized with a microcontroller, thereby lowering the manufacturing costs and energy consumption.

The presented design applied as the emission current or the trap current controller in an embedded system could be useful in many measuring instruments with an electron impact gas ion source where a stable ion current and high repeatability of measurement results are needed. Additionally, in our opinion, part of the control system concerning only the feedforward control algorithm of the electron accelerating voltage can be used with an original vacuum gauge [27] where the emission current is a measure of cesium vapor pressure; however, further experiments are recommended.

## 4. Conclusions

Basic investigations concerning triple-input, double-output electron beam current and accelerating voltage control systems were carried out. The results of the investigations show that the feedback voltage that is directly proportional to the electron beam current and the cathode heating voltage obtained during PID running and used for feedforward control of the electron accelerating voltage allows for independent stabilization of the electron beam current and accelerating voltage. The emission current closed-loop system eliminates the need to annealing the cathode, makes the emission current more resistant to vacuum pressure changes, and offers easy monitoring and configuration. The presented digital control system that uses a PC and the LabVIEW software is lab-friendly; however, it can be easily implemented in an embedded system, making it a suitable sub-component of a low-cost MEMS mass spectrometer device–universal gas sensor, ensuring high quality of the ionizing electron beam.

## Figures and Tables

**Table 1:** Control systems comparison.

	Presented Control System	Previous Control System [21]
Controller hardware platform	PC	µC
Programming language	G (LabVIEW)	C
Control of emission current	Yes	Yes
Control algorithm	PID, gain scheduling	PID, gain scheduling
Feedback signal transferring from the controlled to the control circuit	Instrumentation amplifier	Current mirror
Average relative standard deviation of emission current	0.021%	0.015%
Control of electron accelerating voltage	Yes	No
Maximum percentage change in electron accelerating voltage	0.011%	Estimated 2.360%

## Data Availability

Not applicable.
